# Automatic extraction of subordinate clauses and its application in second language acquisition research

**DOI:** 10.3758/s13428-020-01456-7

**Published:** 2020-09-01

**Authors:** Xiaobin Chen, Theodora Alexopoulou, Ianthi Tsimpli

**Affiliations:** 1 Universität Tübingen, Europastr. 6, 72072 Tübingen, Germany; 2 University of Cambridge, Cambridge, UK

**Keywords:** Subordinate clause extraction, Text analysis, Second language acquisition

## Abstract

Clause subordination is an important linguistic phenomenon that is relevant to research in psycholinguistics, cognitive and behavioral sciences, language acquisition, and computational information retrieval. The paper presents a comprehensive tool called AutoSubClause, which is specifically designed for extracting subordinate clause (SC) information from natural English production. Using dependency parsing, AutoSubClause is able to extract not only information characterizing the three main types of SCs—complement, adverbial, and relative clauses—but also information regarding the internal structure of different clause types and their semantic and structural relations with elements of the main clause. Robustness testing of the system and its underlying dependency parser Stanford CoreNLP showed satisfactory results. To demonstrate the usefulness of AutoSubClause, we used it to analyze a large-scale learner corpus and investigate the effects of first language (L1) on the acquisition of subordination in second language (L2) English. Our analysis shows that learners from an L1 that is typologically different from the L2 in clause subordination tend to have different developmental trajectories from those whose L1 is typologically similar to the L2. Furthermore, the developmental patterns for different types of SCs also vary. This finding suggests the need to approach clausal subordination as a multi-componential construct rather than a unitary one, as is the case in most previous research. Finally, we demonstrate how NLP technology can support research questions that rely on linguistic analysis across various disciplines and help gain new insights with the increasing opportunities for up-scaled analysis.

## Introduction

A typical *clause* is a sequence of words that includes both a subject and a verb and is contrasted to a *phrase* which does not contain both. For example, in (1), *the door opened* and *because the man pushed it* are both clauses, while *the door* and *pushed it* are phrases.

1. The door opened because the man pushed it.

Clause subordination links two clauses so that one, the subordinate clause (SC), is syntactically dependent on and embedded in the other, the main clause (Cristofaro, 2005). A defining characteristic of a SC is its dependency on another clause, which means that it cannot stand by itself. It has to be embedded into another clause and functions as a constituent of that clause. Hence, the clause *because the man pushed it* in (1) is an SC functioning as an adverbial of the sentence, providing information on the cause of the event described by the main clause (*door opening*). Syntactically, an SC is at a lower level in the overall structure than the clause it is subordinate to (Aarts, 2006). Depending on the function an SC plays in the main clause, three types of (SC)s can be identified: complement, relative, and adverbial clauses, exemplified below.

(2) Complement clauses (CCs):
  - I hope *that she will win*.
  - I heard *that she was leaving.*
  - The news *that she is quitting her job* was shared in no time.
  - I wonder *who they will choose in the end*.
(3) Relative clauses (RCs):
  - The guy *that you met* is my ex-husband.
  - It’s a place *where people tend to go on hot days.*
  - A person *who likes books* would love this job.
(4) Adverbial clauses (ACs):
  - I entered the house *as she was leaving.*
  - *When I arrived*, they were already gone.
  - He loved her *even though he could not say why.*
  - We chose chocolate cake *because all the kids love chocolate.*

We will return to a more detailed description of the different types of SCs, but the short list of examples above illustrates the variety of structures and the semantic relations subordination encodes. It is, therefore, not surprising that clause subordination has been widely researched in a number of disciplines, including linguistics, psycholinguistics, cognitive and behavioral sciences, and language acquisition (e.g., Baten & Håkansson 2015; Ozeki & Shirai 2007; Grodzinsky et al., 1999; Divjak 2017; Müller & Penner 1996; Comrie 2002; Gayraud & Martinie 2008; Kyle & Crossley 2018). The reasons for its popularity in the different fields is not only because of the potential linguistic complexity involved in realizing clause subordination, but also because of the higher cognitive demand resulted from such linguistic complexity on the part of the language user. This makes it especially interesting for research on people with limited or impaired linguistic and/or cognitive competence, such as beginning learners of a second language (L2), aphasia patients, and low-literacy speakers of the first language (L1).

Clause subordination has been widely employed to inform theory and practice in second language acquisition (SLA). For instance, researchers have used subordination ratios, i.e. the percentage of SCs to all clauses or number of SCs per sentence, as linguistic complexity measures to assess text readability, benchmark proficiency, and promote proficiency development (Wolfe-Quintero et al., 1998; Lu, 2010; Chen & Meurers, 2017; Vajjala & Meurers, 2012; Norris & Ortega, 2009; Housen et al., 2012) and found the measures to be highly effective for the purposes of their research. Clause subordination is also interesting to the more engineering subject of information extraction. Example studies include the automatic extraction of RC for biomedical text simplification (Peng et al., 2012) and the extraction of specific information from clause analysis for political discourse analysis (van Atteveldt et al., 2017).

The pervasive application of clause subordination in the various fields mentioned above indicates a clear demand for dedicated tools to automatically extract SCs and their related information from language productions, especially in fields where large amount of data need to be analyzed. For example, in a study to investigate the developmental trajectory of RCs among L2 English learners, Alexopoulou et al., (2015) used the C&C Combinatory Categorial Grammar (CCG, Clark and Curran 2007) parser to analyze over 2.3 million sentences written by thousands of learners from five L1 backgrounds. Manual analysis of such big amounts of data would not have been possible without the help of automatic tools.

A number of existing tools offer automatic analysis of SCs by using natural language processing (NLP) technologies (e.g., Dornescu et al., 2014; Lu 2010; Chen & Meurers 2016; Manning et al., 2014; Kyle 2016). For example, the Tool for the Automatic Analysis of Syntactic Sophistication and Complexity (TAASSC, Kyle^2016^) is a recent development that is capable of calculating SC-related linguistic complexity measures such as “ACs per clause”, “subordinating conjunctions per clause”, “clausal subjects per clause” and so on. TAASSC also includes all the general subordination measures from the Syntactic Complexity Analyzer (SCA, Lu 2010) like “dependent clauses per clause” and “dependent clauses per T-unit”. Although these tools have been widely used in research involving linguistic complexity analysis and proved very useful, their output are usually calculated statistics of subordination complexity, rather than the more fine-grained information of individual SC. TAASSC does offer to output NLP processed texts, but additional tools such as brat^1^ and Tregex^2^ are required to visualize or further extract individual SC texts and their related information. This limits the usefulness of the tools for some researchers. For example, SLA researchers who want to inspect L2 learners’ actual use of subordinate conjunctions in RC would find it difficult to obtain a sample of RCs from the learner corpus under investigation with existing tools. The Biber Tagger (Biber, 1988) is another tool capable of extracting various linguistic features and finer-grained SC information such as RCs and their grammatical functions in a sentence (at objects/subject position), CCs and the semantic group of words controlling them as was demonstrated in Biber et al., (2004). Unfortunately, the tools reported in the studies are not publicly available. Besides, there has been no tools to synthesize all the information individual tools can extract. It is therefore time consuming and error prone for researchers to obtain results from multiple sources or tools, while research teams necessitate a specialist to be able to extract more specific information.

What are the optimal design features of a comprehensive tool to support research? From a conceptual point of view, a comprehensive tool for SC extraction should be able to not only identify the three types of subordination, but also provide comprehensive information regarding the internal structure of SCs, the semantic relations established with elements of the main clause, the level of embedding and other aspects that we detail in the next section, where we present the key elements that are relevant to SC analysis. A tool that can go beyond the coarse measures of subordination and provide rich information about a range of linguistic features marking the syntactic function and semantic contribution of SCs to the sentences they are embedded in is very useful for research areas that rely on high levels of linguistic analysis. It is especially useful for studies on L1/L2 development, human sentence processing, and information retrieval.

On a practical level, such a tool should be easy to use and be open to the scientific community so as to foster collaborative development. To meet these needs, we developed the AutoSubClause that makes use of automatic dependency parsing in NLP and rule-based extraction of information from the parsed semantic graphs. In what follows, we will first describe the information AutoSubClause is capable of extracting from English texts and some technical details of the system. Then an example case study in which the system is used to study the effects of L1 on the development of L2 English subordination will be reported. We conclude by making AutoSubClause publicly available and open source.

## Information on clause subordination

SCs are categorized according to their relationship with the main clause. An SC can function as a complement of the main verb, a modifier of main clause phrases like a noun phrase (NP), or an adverbial modifier of the main verb or the main sentence. These three functions characterize the three major types of SCs, namely, CCs, RCs and ACs. In all cases, subordination is typically marked by a special item introducing the SC, a *subordinator* like *that, when, if, etc*. Let us consider in more detail the three main types of SCs.

CCs function as arguments of the main verb, subject or object (Noonan, 2006). In (5-a), *that Zeke eats leeks* is selected as a complement of the main verb *knows* and functions as its object, therefore, an object CC. In (5-b), the same clause is the subject of the sentence, hence a subject CC.^3^

(5)
  - Zelda knows *that Zeke eats leeks*.
  - *That Zeke eats leeks* is surprising.
  A CC can also be selected by a noun as in (6).
(6)
  - The news *that Zeke eats leaks* was surprising.
  - The belief *that Zeke eats leaks* made farmers take extra measures.

A common subordinator for CCs’ is the complementizor *that*, an element without semantic content, which in fact, may be omitted in cases like (7).

(7) I thought *(that) you were gone.*

Verbs like *wonder*, *ask*, *want to know* tend to take indirect questions as complements. There is a variety of subordinators introducing indirect questions, like interrogative *wh*-pronouns or the complementizers *if* or *whether* as illustrated in the examples in (8).

(8)
  - I am curious to find out *who got the job*.
  - I don’t know *if she can make it tonight.*
  - She asked *where to put the boxes.*
  - I wonder *whether she will be able to cope.*
  - I’d like to know *when she left*.

AutoSubClause identifies CCs and further determines whether the clause functions as the subject or object of the verb or a noun.

The second major type of SC is *adverbial clauses*. Adverbial clauses as in (4) usually modify the event denoted by the main verb, providing information regarding the temporal order of the events of the main and subordinate clause, why, how or where the main event took place, as well as various discourse relations as for example in (4-c). Conditionals as in (9-a) or temporal sentences as in (9-b) are further examples.

(9)
  - She won’t do it *unless you ask her yourself.*
  - *Before you enter the room*, please clean your shoes.
  - *When you enter the room*, please follow the signs.
  - She helped him go up the stairs, *so that he could enjoy the view.*

A variety of subordinators introduce ACs and typically convey some basic meaning that is relevant for the semantic relation with the main clause. Thus, the subordinator may signal that the AC is contributing information about time (*when*, *before*, *after*, etc.), condition (*if*, *unless*, *provided*), reason (*because*, *since*, *given*), purpose (*so*, *to*), or result (*so*, *such*) of an action. Syntactically, adverbial clauses are adjuncts to the main verb or clause.

The final major type is relative clauses. Relative clauses typically modify a noun in the main clause, the *head noun*, and may be introduced by a wh-pronoun (10-a), the complementizor *that* (10-b), or nothing at all (10-c). Relative clauses can also be introduced by a *wh* element with adverbial meaning, indicating place (11-a) or time (11-b).

(10)
  - The headmaster, *who we met on a trip to Scotland,* is a very nice and warm person.
  - The man *that was sitting in the front row* is the president’s husband.
  - I liked the book *you gave me.*
(11)
  - It’s a nice place *where you can relax with your family.*
  - It was the day *when I realized that life is not always fair.*

Free relative clauses do not modify a noun of the main clause, rather, they function directly as complements.

(12)
  - I will eat *what you eat*.
  - *Whoever solves the puzzle,* will be our first guest of the show.
  - I’ll go *wherever life takes me*.

The overview presented so far shows the rich structural and semantic relations encoded through subordination. Crucially, the correct classification of SCs cannot be achieved out of context and needs to take into account various pieces of information, e.g., the subordinator, if other phrases function as complements or adjuncts of the main verb, etc. For example, a sentence introduced by *if* could be a conditional AC or an indirect question functioning as a CC of the main verb. Similarly, a sentence like *what you eat* could be a free relative clause or an indirect question, depending on the main verb. An SC following a noun and introduced by *that* could be a complement clause (6) or a relative clause (10-b).

The categorization presented above is based on the relation of SCs with the main clause. SCs can also be categorized according to their internal properties, some of which are of particular interest to linguists and psycholinguists.

One important property of SC is finiteness, that is, the presence of tense and in some cases agreement morphology on the main verb. Thus, the verb *left* in the SC of (13-a) is a finite form marked for past tense, while in (13-b) the non-finite form *leaving* is used. Unlike finite clauses, non-finite clauses typically do not have an overt subject as in (13-b), where the subject of *leaving* is the same with the main clause subject, *I*. Subordination can, thus, be signaled by desententialization (Lehmann, 1988; Aarts, 2006), in which case the subject of the SC is missing and a nonfinite form of the verb is used, as also exemplified by (14). The clause *to emigrate to Australia* in (14-a), lacks an overt subject, since the subordinate subject is co-referential with the main clause subject—it is *I* who wants to emigrate to Australia.

Overt subjects of non-finite clauses are exceptional in the accusative as in (14-b,c), which signals that the subordinate subject is not co-referential with the main clause subject.

(13)
  - *Before she left*, she made sure all windows were closed.
  - I had something to eat *before leaving*.
(14)
  - I want *to emigrate to Australia*.
  - They wanted *him to emigrate to Australia.*
  - The made *her leave.*

The acquisition of finiteness and its interaction with subordination and subjecthood is central to research on language development (Guasti, 2016; White, 1991; Owen and Leonard, 2006; Steel et al., 2016; Yang, 2014). For instance, Owen and Leonard (2006) found that children between 5 to 8 years of age were more accurate in using nonfinite CCs than finite ones in their L1. However, after analyzing the use of nonfinite clauses in the academic writings of Chinese learners of English, Yang (2014) discovered that the learners’ ability to use nonfinite clauses was correlated with their L2 proficiency.

An SC can also be verbless, as *her car a wreck* in (15).

(15) Sally had to walk home, *her car a wreck*.

Since nonfinite and verbless clauses cannot function as complete sentences, they always need to be embedded into other clauses and be subordinate to them.

Another piece of information relevant to clause subordination is the level of embeddedness (LoE), which denotes how deep the SC is from the top-level main clause of the sentence. All examples of SCs we have considered so far have an embedding depth of one because they are structurally one level below the main clause. By contrast, the clause *who Uncle Bill had hired* in (16) is embedded two levels below the main clause, as it is embedded in *that the maid who Uncle Bill had hired* which has a LoE of one.

(16) My brother opened the window *that the maid* *who Uncle Bill had hired* *closed*.

LoE is interesting for L2 development. For example, Geertzen et al., (2014) provided examples showing that Brazilian learners of English tend to produce SC that are embedded deeper, while examples by their Chinese counterparts appear embedded only under the main clause.

To summarize so far, AutoSubClause extracts and identifies information regarding the relation of an SC with the main clause, as well as further information and features. Thus, for all three types of SCs, AutoSubClause identifies the clause text and determines the clause type; it further determines the finiteness of the clause and its LoE. If present, the system extracts the subordinator (a complementizor, relativizor, or subordinating conjunction) as well.

For ACs, it extracts the meanings of the ACs additionally, such as reason, time, place, purpose, or manner and so on.

Let us now consider RCs in more detail. In an RC the head noun appears at the beginning of the clause, but might realize various grammatical functions: it can be a subject as in (17-a), an object as in (17-b), an indirect object/complement of a preposition as in (17-c) or a possessor as in (17-d).

(17)
  - the composer *that wrote this masterpiece*...
  - the composer *that Helen invited to her wedding*...
  - the composer *that Helen spoke to*...
  - the composer *whose masterpiece Helen played at her wedding*..

The role the head noun plays in the RC is of particular interest to language typologists, psycholinguists, and language acquisition scientists. Keenan and Comrie (1977) proposed the noun phrase accessibility hierarchy (NPAH), which postulates that the ease of relativization is determined by the grammatical role of the head NP modified by the RC. NPAH is expressed as the hierarchy *subject >direct object >indirect object >oblique >genitive >object of comparison*. Typologically speaking, if a language allows relativization of an NP at a position in the hierarchy, it must also allow relativization at all the higher positions. There is psycholinguistic evidence that RC involving relativization from lower positions in the hierarchy are harder to process (Gibson, 2000) while subject relatives are strongly preferred in early productions in both L1 and L2 acquisition (Kim & O’Grady, 2016). The NPAH has been widely researched in both L1 and L2 acquisition (Ozeki & Shirai, 2007; Comrie, 2002; 2007; Kanno, 2007; Yip & Matthews, 2007; Eckman, 2007; Kim & Christianson, 2017; Hyltenstam, 1984). Given the prominence of this question in research, the AutoSubClause system extracts the grammatical role of RCs in both the main and subordinate clauses.

A further property of the head noun that has attracted attention is its animacy. Head noun animacy is important for information retrieval (Orǎsan & Evans, 2007; Jahan et al., 2018) but is also of interest to language acquisition (Ozeki & Shirai, 2007; Jeon & Kim, 2007). Shirai and Ozeki (2007) suggested that there might be an interaction between the grammatical role of the head noun (i.e., subject, direct object, indirect object, etc.) and its animacy when it comes to the acquisitional sequence or learner preference. They cited evidence from Ozeki and Shirai (2007), who found that L2 Japanese learners prefer using subject RCs with animate heads and direct object RCs with inanimate heads (Shirai & Ozeki 2007, p. 160). Alexopoulou et al., (2015) reported that Chinese, German, and Russian learners of English avoided the complementizor *that* with animate heads, in contrast to Brazilian, Mexicans, and Italian learners.

Beyond acquisition, Jahan et al., (2018) point out that the semantic property of NP animacy is a useful property for a number of NLP-related tasks such as word sense disambiguation, semantic role labeling, and coreference resolution. Therefore, when extracting information on RCs, our system also detects the animacy of the head noun the clause modifies.

A final aspect of RCs is the distinction between restrictive and non-restrictive relatives. A restrictive RC is used to (uniquely) identify an individual in the discourse. Thus, in (18-a), the RC is used to help identify which of the many women in the discourse is the president’s wife. By contrast, the RC in (18-b) and (18-a) is not used for identification, but only to provide additional information on the president’s wife.

(18)
  - Who is the president’s wife? *The lady that you saw visiting the exhibition yesterday.*
  - The president’s wife, *who I had met at the exhibition the night before*, entered the room.
  - John, *who is a good teacher*, got a new job.

(18-a) is a restrictive relative clause while (18-b), (18-c) are non-restrictive RCs. A restrictive relative clause restricts the domain of relativation, identifying the referent of the head noun, in (18-a) which lady is referred to(Keenan and Comrie, 1977; Keenan, 1985). A non-restrictive relative provides further background information on the head noun whose reference does not need to be identified by the RC (Nikolaeva, 2006).

To sum up, for all SCs, our system extracts the clause type, its finiteness, subordinator, and LoE. Additionally, for CCs, the system determines whether the clause is a subject or object complement. For ACs, the function of the clause is identified. Restrictiveness of the RC, the head noun and its animacy, as well as the grammatical roles it plays in both the main and relative clauses can also be extracted by the system.

## Technical details and evaluation

**Fig. 1:**
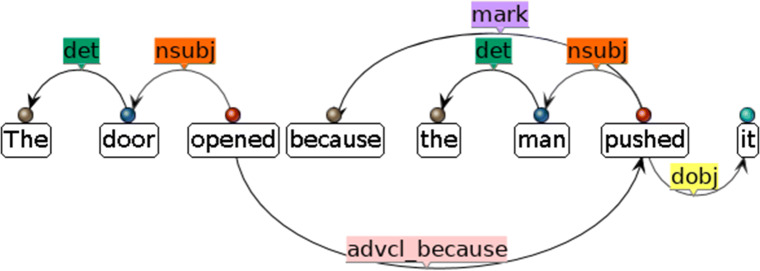
Dependency graph showing dependency parse result from the Stanford CoreNLP Dependency Parser

The AutoSubClause system extracts SCs from the dependency parses of sentences. A dependency parse is an analysis of the syntactic structure of a sentence based on a dependency grammar. There is a variety of dependency grammars, but the essential idea is that the syntactic structure of a sentence “consists of words linked by binary, asymmetrical relations called dependency relations (or dependencies for short)” (Kübler et al., 2009, p. 2). A dependency relation connects two separate words, with one being the *governor* (or *head*) and the other the *dependent* (or *modifier*). The relation between the word pair is marked with the type of dependency. Figure 1 shows the dependency parse of example sentence (1), which is represented and can be stored in the computer as a semantic graph.^4^ Each directed arc in Fig. 1 represents a dependency relation between a pair of words. The starting point of an arc is the governor of the relation and the ending point the dependent. The starting point of the whole graph is also called the root of the sentence, which usually is the main verb in the main clause. The arc label marks the type of relation between the governor and the dependent. For example, the words *man* and *pushed* are connected by an arc marked as *nsubj*, which means *man* is governed by the verb *pushed* and is the nominal subject (*nsubj*) of the verb. The set of relations allowed in dependency parsing is dependent on the grammar. The example parse shown in Fig. 1 uses the Universal Dependencies (UD, de Marneffe et al., 2014), a grammar that aims at creating a cross-lingual formalism of dependency grammar.

**Fig. 2:**
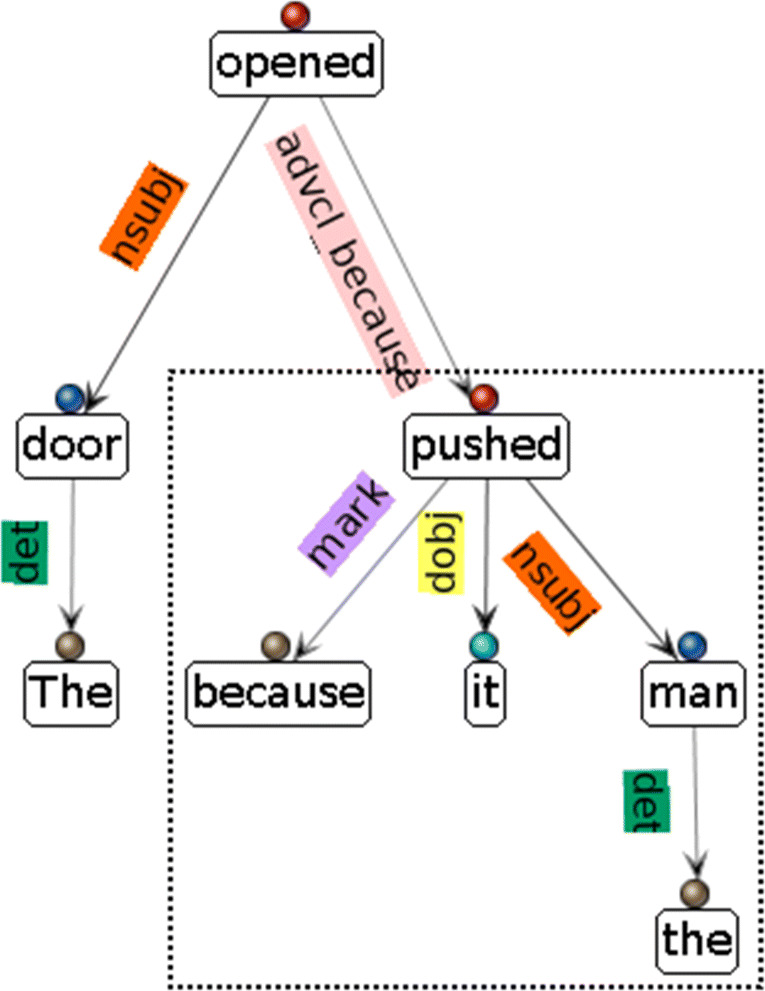
A semantic parse tree from which the SC can be extracted by extracting the span of the tree rooted at the dependent of a subordinating relation (*pushed* of the *advcl* relation)

AutoSubClause uses Stanford CoreNLP (Manning et al., 2014), which uses the UD, for dependency parsing. CoreNLP (hence also UD) is able to identify all three types of SC relations. Adverbial clauses are marked with the *advcl* or *xcomp* relations, CCs as *ccomp*, *csubj*, or *csubjpass* relations, and RCs as *acl* relation between the verbs of the main and subordinate clauses.^5^ The dependency relation holds between the main clause verb, which is the governor, and the subordinate clause verb, which is the dependent. The main verb is the root of the parse tree.^6^ As a result, the way to extract an SC is to extract the span of the parse tree starting from the clause root, as is shown by the dotted box in Fig. 2. A dependency parse tree like Fig. 2 is a computational data representation of the semantic relations between the words in a sentence. The output of CoreNLP’s dependency parser is general purpose NLP pre-processing results with which further operations such as information retrieval and SC extraction can be conducted. In essence, the output of CoreNLP contains signaling information of clause subordination which AutoSubClause uses to extract the actual SCs and their related information.

As was discussed in Section 2, an automatic SC extraction system should not only be able to extract the clause text, but also the type of the SC, its finiteness, subordinator if one exists, as well as LoE. AutoSubClause identifies clause types by looking at the label of the subordinate relation. It then goes on to identify the finiteness of the SC by applying a number of rules. For example, if the subordinate root or main verb of the SC directly follows the infinitive marker *to* or a preposition, the SC is nonfinite. If it is a gerund and has an *aux* relation (auxiliary, e.g., a modal auxiliary, or a form of “be”, “do”, or “have”) with another word, the clause is finite, otherwise nonfinite. For example, in *is leaving* and *have been reading*, the gerunds *leaving* and *reading* govern the auxiliaries *is* and *have*, hence the verbs are finite.

The subordinator is identifiable by finding a *mark* relation from the clause root. The *mark* relation is defined as “the word introducing a finite clause subordinate to another clause” by the UD. For example, in Fig. 1, *pushed* is “marked” by *because*, which is the subordinator, or subordinate conjunction, of the SC. For RCs, a *ref* relation, which is straightforwardly defined as “the relative word introducing the RC modifying the NP”, is sought instead. Null values would be given if no subordinators are identifiable.

The LoE is calculated as the maximum distance to a least common ancestor of the sentence and SC roots in the dependency tree. For the parse tree of example (1) shown in Fig 2, the sentence root, which is also the root of the parse tree, is the word *opened*. The root of the SC is *pushed*. As a result, the SC is embedded one level down the main clause because the maximum distance to the common tree rooted at *opened* for the sentence and SC roots is one.

**Fig. 3:**
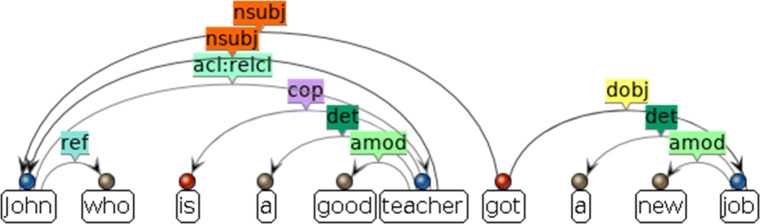
The dependency parse of a nonrestrictive RC

Besides information common to all types of SCs, AutoSubClause also extracts type-specific information. For CCs, the system determines the complementary function of the SC by simply looking at the relative positions of the governor and the dependent of the complement relation. Since English is a subject-verb-object language, if the governor is in front of the dependent, the CC is considered an object complement of the sentence. Otherwise it is a subject complement.^7^ For adverbial clauses, the system determines the semantic function of the clause using the subordinator. It decides whether the SC is expressing a relation of time, place, condition, reason, or manner, etc. The distinction between restrictive and non-restrictive relative clauses is a discourse-semantic one, and, therefore, hard to capture with information provided by a dependency grammar. There are, nevertheless, some structural corelates of this semantic distinction that AutoSubClause utilizes as a first approximation to classifying relative clauses into restrictive and non-restrictive ones. AutoSubClause first decides whether the clause is restrictive or not, by applying a series of rules. For example, an RC with a zero relativizer can only be a restrictive one. If the head noun of the clause is a personal pronoun or proper noun, the clause is most likely nonrestrictive as in (18-c). Besides restrictiveness, the system also identifies the head noun and its animacy. The head noun is extracted from the governor of the *acl* relation (Fig. 3). We used dictionary lookup to determine the animacy of the head noun, although a more accurate method is probably to use statistical models (Orǎsan & Evans, 2007; Jahan et al., 2018).

**Fig. 4:**
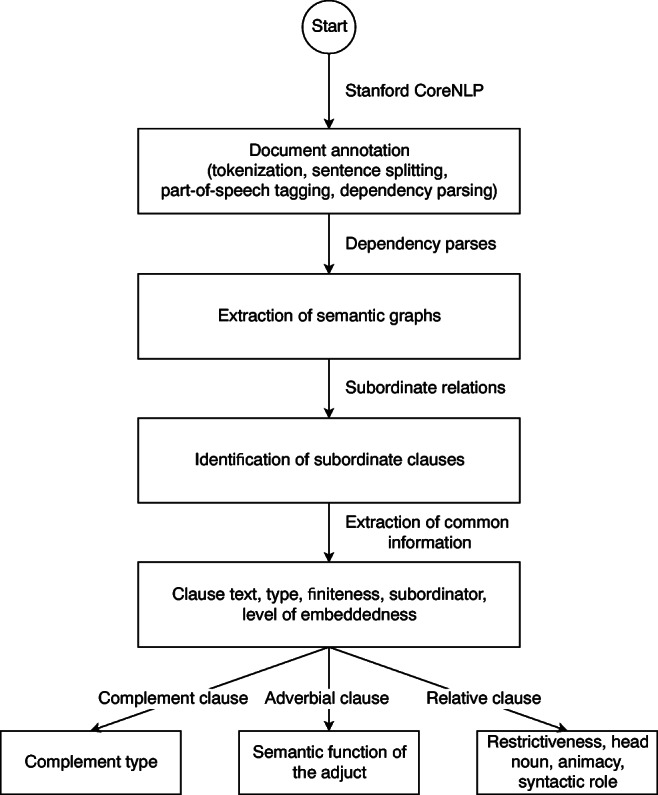
Pipeline for extracting information on different types of SCs

As discussed in the previous section, an interesting piece of information is the syntactic role the head noun plays in both the main and subordinate clauses. AutoSubClause identifies this information by looking for dependencies where the head noun is a dependent. Combining with the position of the governors of these dependencies, the system is able to determine the role of the head noun such as subject, direct object, indirect object, preposition complement, or appositive in both the main and relative clauses. Figure 4 shows the pipeline for SC information extraction used by the system.

The accuracy of the AutoSubClause system depends mostly on the accuracy of the dependency parser because all SC information is extracted from the dependency parses. Chen and Manning (2014) evaluated the CoreNLP dependency parser used in our system with a part of the English Peen Treebank (PTB, Taylor et al., 2003) consisting of 44,389 words. The PTB is a human-annotated corpus of texts produced by native speakers from sources like the Wall Street Journal and the Brown corpus. It is widely used as a gold standard for training and testing NLP tools. Chen & Manning reported their evaluation results with two metrics: unlabeled attachment score (UAS) and labeled attachment score (LAS). The former refers to the proportion of words that are assigned the correct governors regardless of the type of relation, while the latter takes into consideration the correct assignment of relation types as well. They reported 91.8% and 89.6% for UAS and LAS, respectively for the CoreNLP dependency parser. This performance placed the parser on the top of the most accurate dependency parsing systems.

The parser also works well with learner texts. Interested in the performance of NLP tools on learner produced texts, Geertzen et al., (2013) evaluated CoreNLP’s dependency parser with a set of 1000 sentences (11,067 words) pseudo-randomly sampled with equal representation of proficiency levels from EFCAMDAT, a large learner corpus consisting of scripts written by thousands of learners from all over the world. They found that the parser was also robust in parsing learner produced texts, with overall accuracy of 92.1% on UAS and 89.6% on LAS. The performance of the parser was better when parsing sentences without learner errors than ones with at least one error. The accuracy difference was about 10% on both UAS and LAS (ibid. Table 4). Geertzen et al., (2013) also listed detailed precision and recall statistics on the performance of the parser on different types of relations (ibid. Table 7). The performance of CoreNLP was quite satisfactory for all the subordinating relations used by AutoSubClause, except for subject clauses (csubj), a type of structure rarely used by L1 and L2 learners. All calculated F scores (harmonious means of precision and recall) are above 89%. The authors attributed the good performance to both the overall simplicity of learner productions and the robustness of the parser to focus on the primary dependency relations so learner errors at the morphosyntactic and semantic levels do not affect the parser’s judgment of the syntactic patterns. Hence they concluded that the CoreNLP tools could also be safely used to analyze learner texts. Similar results were reported by Huang et al., (2018). As a result, given the fact that the SC information extracted by AutoSubClause is from the dependency parses output by CoreNLP, the performance of our system would also be reliable. This is confirmed with a validation study reported in the next paragraph.

In order to confirm the system’s performance on real-life data, a validation study was conducted with both authentic and learner produced data. A total of 2,212 sentences were sampled from a corpus of authentic texts and analyzed with AutoSubClause. A native speaker of British English and linguist was asked to confirm the correctness of the results produced by the system. The overall performance of the system on authentic sentences was *F*1 = .938 with precision and recall values of .895 and .985, respectively. For L2 data, only RC extraction was evaluated. A corpus of 5,908 sentences produced by L2 English learners were randomly sampled from EFCAMDAT. AutoSubClause was used to extract RCs from these sentences. One of the authors of the paper who is an experienced linguistics researcher manually annotated the extracted results and confirmed the robustness of the system. On L2 data for RC extraction, the overall performance of AutoSubClause was *F* = .875 with a precision of .861 and recall of .889. These validation results reflect the high performance of the underlying NLP tools reported in the other studies described in the previous paragraph.

## L1 effects on the development of L2 subordination: a use case of autosubclause

In this section, we present a study in which AutoSubClause is used to analyze a large L2 English corpus consisting of scripts written by thousands of learners from all over the world participating in an online English course. The aim of the study is to investigate the effects of L1 on the development of L2 subordination. In what follows, we will first have a brief review of literature to justify the study. Then the data and analysis results will be presented before conclusions are drawn.

### Research on L2 Subordination

There has been extensive research on the acquisition of subordinate clauses in both L1 and L2 acquisition (e.g., Müller and Penner 1996; Baten & Håkansson 2015; Gass & Lee 2007). Previous studies approach subordination both as an acquisitional target that has its own developmental characteristics and sequence and as a descriptor of learner language to gauge proficiency, describe performance, and benchmark development, especially within the framework of complexity, accuracy, and fluency (CAF, Housen et al., 2009, 2012; Ortega 2012) which is often applied in task-based SLA research. These studies rely on subordination ratios to measure L2 complexity as valid indicators of L2 proficiency showing a linear developmental trajectory (Wolfe-Quintero et al., 1998). Subordination ratio is calculated as the percentage of SCs to all clauses or number of SCs per sentence. In fact, in a comprehensive review of L2 complexity research, Norris and Ortega (2009) found that all 16 studies they reviewed used subordination ratios without exception.

This extensive body of research in L2 subordination tends to view subordination as a unitary construct of global syntactic complexity (Lambert & Kormos, 2014). For example, Baten and Håkansson (2015) examined the development of subordinate clauses in L2 German and Swedish from the perspectives of subordination ratios and subordinate clause word order, but not the possible differences between different types of subordinate clauses. Other studies (e.g., Flynn et al.,^2004^; Ozeki & Shirai 2007; Comrie 2002; Kanno 2007) focused on RC alone. However, given that complementation, relativization and adverbial modification involve very different structural and semantic operations, there are good reasons to consider a more detailed anatomy of the subordination construct, as was already pointed out by Lambert and Kormos (2014). L2 researchers have long been calling for more fine-grained analysis of linguistic complexity in general and of subordination complexity in particular (Biber et al., 2004; Biber et al., 2011; Bulté & Housen, 2012; Kyle & Crossley, 2018). Results from their studies also showed that fine-grained syntactic complexity measures account for more variance in L2 writing quality or development than the more traditional large-grained syntactic complexity indices (Biber et al., 2011; Kyle & Crossley 2017, 2018). Therefore, the use of AutoSubClause to research L2 subordination development fits well into this line of research.

A further feature of previous research is that measurements rely on small sample sizes, primarily due to the labor intensiveness of linguistic data collection and analysis. For example, Baten and Håkansson (2015) collected speech and written data from nine L2 German and 19 L2 Swedish learners with six interviews and three written essays, respectively. Ozeki and Shirai (2007) focused on the RCs produced by 90 learners of L2 Japanese, but still only managed to extract 1,005 RCs for analysis. Alexopoulou et al., (2015) was one of the few studies that used NLP to analyze large amount of learner data for studying the development of RCs. They extracted RCs from a 33-million-word corpus with the CCG parser. However, due to the limitation of the parser, zero RCs (e.g., the girl *I saw*) were excluded, giving an incomplete account of the construct.

Last but not least, few studies have investigated the detailed aspects of subordinate clauses such as the use of subordinators, the LoE, the semantic function of ACs, the role the head noun of a RC plays in both the main and subordinate clauses and so on. The current study tries to fill some of these gaps by using the AutoSubClause system. It is also a demonstration of how the system can be used to tackle SLA questions.

We approach the problem from the perspective of L1 effects on the development of L2 subordination, which has received special attention in recent SLA research (e.g., Ozeki and Shirai 2007; Flynn et al., 2004; Yip & Matthews 2007; Alexopoulou et al., 2015). To illustrate the usefulness of our tool, we investigate the following empirical questions:

- Are there L1 effects on the overall development of L2 subordination? If yes, what are the effects?
- Are there clause type effects when it comes to L2 learners’ use of SCs? If yes, what are the effects?

To answer these questions, we will look at two factors that are potentially linked to subordination usage across proficiency levels: the learner’s L1 and types of SCs.

### The data

A balanced sample of 31,040 scripts from 16 proficiency levels (1,940 scripts/level) was drawn from EFCAMDAT (Geertzen et al., 2013), a publicly available corpus consisting of 71.8 million words (1.2 million scripts) written by over 174k learners from 188 countries.^8^ The learners were all participants of an online course offered by the international English language school Education First. The sampled corpus consists of 3.8 million words and 240k sentences. Only proficiency levels were balanced when sampling. Our analysis focused on the four groups of learners representing major L1 backgrounds in the corpus: Brazilians, Chinese, Russians, and Japanese. It should be noted that EFCAMDAT uses national language as a proxy to L1 so the country from which a learner accesses the online course is taken as the learner’s L1 being the national language of that country. National language is nationality combined with country of access (e.g., a Japanese national accessing the system from Brazil is excluded from the data). AutoSubClause was used to extract SCs from the sampled corpus. Although the system is capable of extracting both finite and nonfinite clauses, only finite clauses are included in the analysis.^9^

### Results

**Fig. 5:**
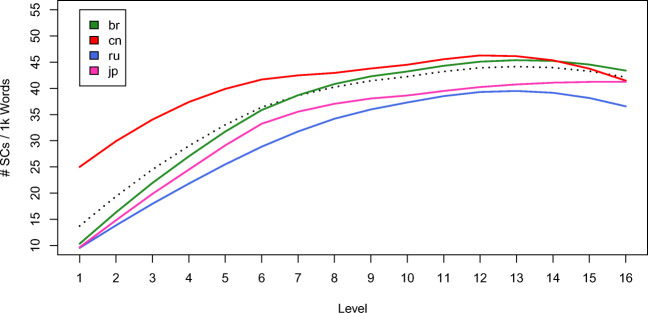
Mean number of SCs per 1k words by proficiency levels for learners with different L1s

A total of 72,222 finite SCs were extracted^10^ from the four target L1 groups in the corpus. Table 1 summarizes the distribution of different types SCs across proficiency levels. In order to compare the usage of SCs across proficiency levels, we calculated the normalized index of number of SCs per 1k words to account for script length effect. Figure 5 shows the development of L2 subordination usage across proficiency levels. The black dotted curve shows the development of all L1 groups, while the colored curves show development of specific L1s. It can be seen from Fig. 5 that overall, L2 English learners use more and more SCs as their proficiency increases. A one-way ANOVA confirmed the observation, *F*(15) = 194.7,*p* < .001. The increasing trend reaches a plateau at around Level 11, which corresponds to B2 in the Common European Framework of Reference (CEFR, Council of Europe, Council of Europe 2001), a finding consistent with Alexopoulou et al., (2017) and earlier findings (Bardovi-Harlig and Bofman, 1989; Perkins, 1980; Scott, 1988). The developmental trend is similar for learners of different L1 backgrounds. However, as the colored curves in Fig. 5 show, learners of different L1s vary in the amount of use of SCs. Chinese learners use consistently more SCs than the other L1 groups, but they also reach a plateau earlier—at around Level 8, or B1 in CEFR. Possible reasons for the leveling off of SC ratio at the higher proficiency levels include the limit of linguistic need to express ideas with subordination and the learners’ ability to use other structures such as complex nominals.

**Table 1:** Number of different types of RCs from different proficiency levels

	Clause type			
Level	Adverbial	Complement	Relative	Total
1	99	328	364	791
2	270	639	532	1441
3	232	807	454	1493
4	978	964	635	2577
5	875	1386	789	3050
6	937	2214	1058	4209
7	1057	2053	1071	4181
8	1416	2159	1103	4678
9	1855	2193	1133	5181
10	1521	2560	1294	5375
11	2009	3113	1812	6934
12	2182	3144	1511	6837
13	1625	2843	1764	6232
14	2130	3064	1839	7033
15	1802	3001	1877	6680
16	1458	2205	1867	5530
Total	20,446	32,676	19,103	72,222

**Fig. 6:**
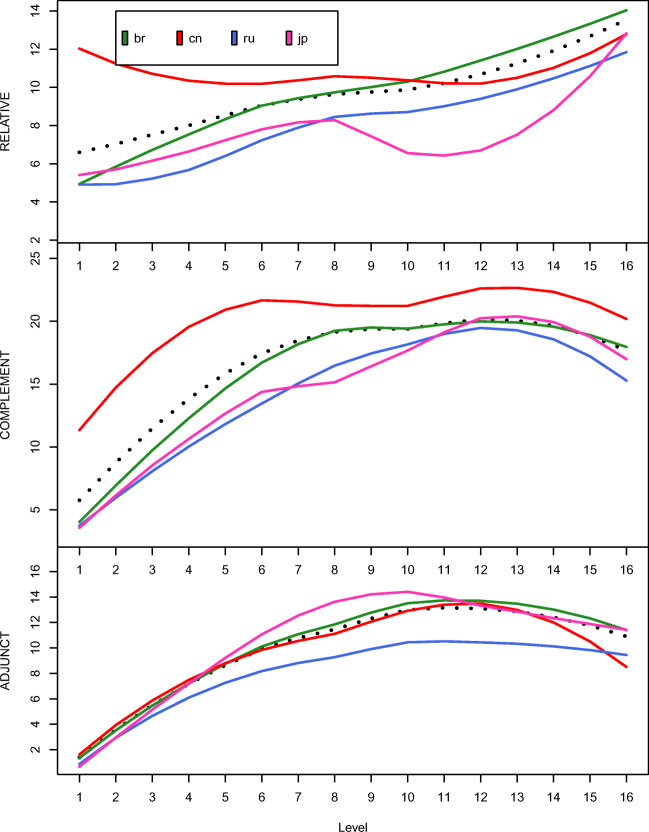
Comparison of the developmental trajectories of different types of SCs from learners of different L1s

Figure 6 compares the developmental trajectories of different types of SCs for different L1 groups. Chinese learners use more SCs in general (Fig. 5). Their more frequent use is mainly driven by CCs at all levels and RCs at the beginning to intermediate levels (top and middle panels of Fig. 6). Russian learners use fewer ACs than the other three groups at all proficiency levels. For the Brazilian learners, as their proficiency improves, the use of RCs keeps increasing throughout. However, the number of CCs and ACs per 1k words remain mostly at the same level after Levels 8 and 10, respectively. The same pattern occurs with the Russian learners, while the Japanese learners resemble more the Chinese learners.

It is beyond our scope to provide an account of the observed differences between different L1s. There are known typological differences between English and the various L1s that might underlie some of these effects. For example, in the cases of RCs, because both Chinese and Japanese contain only prenominal modifiers but English RC is postnominal and requires a relativizer, Chinese and Japanese learners of L2 English may find it difficult to learn and use English RCs. On the other hand, Brazilian Portuguese and Russian both allow postnominal RCs introduced by relativizers, hence it might be easier for learners of these L1s to learn and use the English RC structure (Schachter, 1974; Flynn et al., 2004). Another source of difference might be how different L1 groups use formulaic sequences. In a corpus coming from an EFL teaching environment, it is possible that learners use subordinate clauses lifted from their input or task-prompts (Wray, 2002; Alexopoulou et al., 2015).

**Fig. 7:**
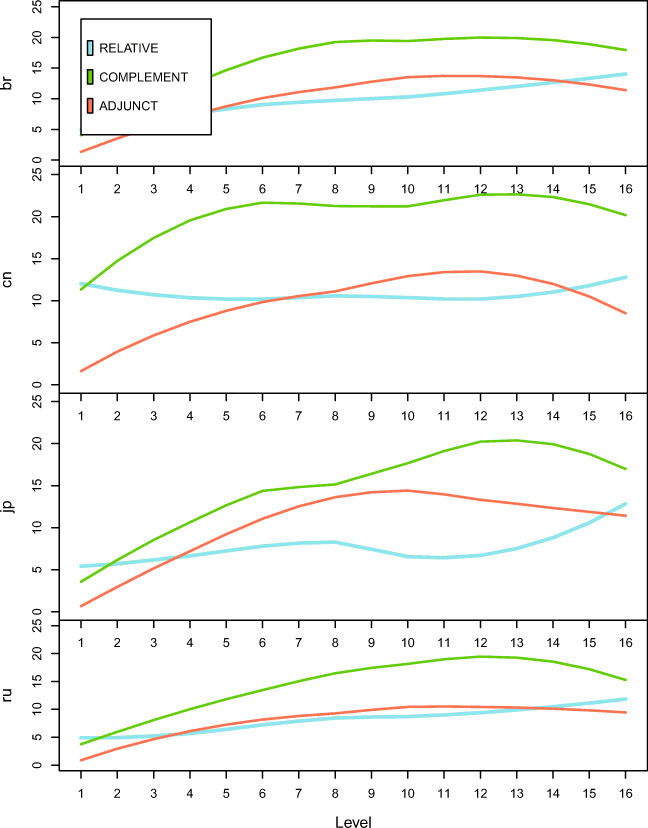
Comparison of the developmental trajectories of different types of SCs by learners of different L1s

The within L1 comparison of the development of different types of SCs (Fig. 7) also shows an uneven development. For all L1 groups, CCs are always the most used type of SCs. For the other two types of SCs, L1 effects are also observed. The Russian learners use approximately the same amount of relative and ACs, while the other L1 groups’ use of these two types of SCs is a function of the proficiency level. At lower levels, the Brazilian, Chinese, and Japanese learners generally use more RCs than ACs. However, as their proficiency develops, more ACs than RCs are observable from the learner production.

These findings reveal that there are L1 and clause type effects on the development of L2 subordination use. Learners whose L1s are typologically closer to the target language in terms of clause subordination tend to have a more consistent and continuous development of subordination usage, while those whose L1s are more different from the L2 would reach a developmental plateau earlier. Besides L1, the learner’s use of subordination across the proficiency levels is also dependent on the type of SCs. Complement clauses seem to be the most natural type of SCs for the learners because their usage keeps increasing throughout with the increase of the learner’s proficiency. RCs pose a challenge for both Chinese and Japanese learners of English, while Russian learners might need more help with ACs. Note that all learners have followed the same lessons and written the same tasks, so the differences could not be due to task effects.

A more detailed analysis of the data is beyond the scope of the current paper. This cursory study nevertheless illustrates how more detailed information on the extracted SCs will help shed more light on the developmental characteristics of L2 English learners from various L1 backgrounds. The previous analysis is also limited in that we did not take into account the effects of formulaic sequences or learning tasks, which have both been found to contribute to SC production (Alexopoulou et al., 2015). Neither did we take into account individual variations within each proficiency level, although it has been found to be important for modeling L2 development (Murakami, 2016). The purpose of the current analysis is to showcase the functionalities of AutoSubClause in analyzing large corpus data. Researchers interested in further exploring related SLA questions are welcome to utilize the tool for more in-depth analysis and modeling. It should also be noted that although the term “developmental trajectory” was used to refer to the trend of subordination usage across proficiency levels, these trajectories are not developmental in the sense of longitudinal development of the learners, either individually or as groups. This is because EFCAMDAT was collected cross-sectionally from learners taking English courses of different levels. The texts produced by the learners were also written responses to different task prompts. As a result, interpretation of the results presented above should be based on full understanding of the characteristics of EFCAMDAT from which we sample our corpus. For a detailed introduction to the corpus please refer to Geertzen et al., (2013).

## Conclusions

Clause subordination is an important linguistic phenomenon that has a number of applications in psycholinguistics, cognitive and behavioral sciences, language acquisition, and computational information retrieval. The automatic extraction of SCs is important for studies that require analysis of large amount of natural language data, which is very common in a lot of modern disciplines. The paper presented a system called AutoSubClause specifically designed for extracting SC information, including type neutral and type specific information. The system is built on top of dependency parsing with CoreNLP, one of the most popular NLP toolkits with state-of-the-art performance. Previous studies have evaluated the parser with both authentic and learner productions and confirmed its robustness. The performance of our system was also evaluated with manual annotation and the results were satisfactory. As an example, the system was used to analyze a large multi-million-word learner corpus with the purpose of investigating the effects of L1 on the acquisition of L2 English subordination. Results of the analysis confirmed the usefulness of the system and showed that the learners’ L1 did affect the usage and development of different types of SCs in the target language. The development and application of the AutoSubClause system shows how NLP technology can be used to meet the linguistic analysis needs from various fields, especially for research that requires up-scaled analysis of large amount of natural language data.

The system currently only supports analysis of English data. The source code is freely available as a Java library on GitHub at https://github.com/ctapweb/AutoSubClause. Future development may focus on multilingual support and the provision of a Graphical User Interface for users with limited programming skills.

## Footnotes

1 http://brat.nlplab.org/

2 https://nlp.stanford.edu/software/tregex.shtml

3 In many syntactic approaches subject clauses are not considered a type of complement; rather, the term *complement* is reserved only for phrases or clauses selected by the verb (as objects Haegeman 1991). This terminological distinction does not affect the present study.

4 In computer science, a graph is a data structure for storing interconnected objects represented as vertices (points) and edges (links between points). In dependency parsing, the edges convey semantic relations between the vertices, hence the resulting graph is called semantic graph. An acyclic connected graph is also a tree.

5 Details about the relation labels and the UD grammar can be found in the Stanford Dependencies Manual or on the UD website at http://universaldependencies.org/en/index.html

6 Strictly speaking, a graph is not necessarily a tree in computer data structure, but CoreNLP’s dependency parser always parses sentences as trees.

7 We do not consider the rare cases of complement inversion such as in “That he could behave so arrogantly, I could never imagine”, although they are also grammatical.

8 These are statistics of the latest version (Version 2).

9 The analysis of infinitival clauses requires analysis of unexpressed arguments that is beyond our scope; descriptively many infinitival clauses might not be considered full clauses.

10 In this paper we do not consider the case of formulaic uses of subordinate clauses, that is, the possibility that learners use SCs lifted from their task prompts that potentially are unanalyzed and, as such, do not reflect true knowledge of SCs. Alexopoulou et al., (2015) present a method for automatic identification of formulaic uses. In their analysis, removing the formulaic uses of relative clauses did not impact on the overall results.
